# Commentary: A crisis in comparative psychology: where have all the undergraduates gone?

**DOI:** 10.3389/fpsyg.2015.01928

**Published:** 2015-12-21

**Authors:** Michael J. Beran, Brielle T. James, Sara E. Futch, Audrey E. Parrish

**Affiliations:** ^1^Department of Psychology and Language Research Center, Georgia State UniversityAtlanta, GA, USA; ^2^Department of Psychology, Wofford CollegeSpartanburg, SC, USA

**Keywords:** comparative psychology, comparative cognition, mentoring, undergraduate students, graduate students

Abramson (2015) has offered an interesting target article, and we are pleased to engage in the debate. Our authors consist of a senior researcher with 20 years of experience working in comparative psychology (MJB), an early career psychologist who just completed her doctoral program in cognitive sciences with a focus on comparative cognition (AEP), a first-year graduate student also in cognitive sciences (BTJ), and an undergraduate student who has worked with multiple species of nonhuman primates in various internship positions (SEF). MJB circulated this article to get reactions from his co-authors given that they presently are heavily invested in comparative psychology at various stages in their careers, to see how they perceive its future.

None of us felt that as undergraduates we needed a course in comparative psychology (formally) to have found our way to being interested in comparative psychology, which was good because none of us had such a course. MJB, for example, took courses in Learning and Behavior and Cognitive Psychology that led him to potential graduate programs in comparative psychology (primarily in areas focused on cognition or psychopharmacology). AEP completed her biology undergraduate degree with very little formal training in psychology, but was introduced to and intrigued by comparative psychology through zoology, animal behavior, primatology, and evolution courses. BTJ noted that she also took the comparative psychology classes that were “hidden” under other names (primate behavior, biological anthropology, etc.), but that for her this still led her to comparative psychology as a graduate student. Thus, we believe that students who go into other specializations are not disengaged with the field of comparative psychology, nor are the psychologists in other specializations incapable of being considered comparative psychologists, if those other specializations recognize the importance of comparative approaches.

We agree with Abramson (2015) that comparative psychology needs to be clearer in its projection to students of what it offers by way of broad skills learned while studying this area. We further note that this same point can be made with translating our work and its implications for the general public. We know that our work has benefits to human society through educational reform, communication theory, behavior modification, and so forth, but we imagine that this clarity of relevance was better made in decades past when, for example, Skinnerian theories and approaches were well known and well integrated into everyday life.

We appreciate Dr. Abramson's concern that comparative psychology not be confused (or worse, equated) with comparative cognition. Comparative cognition draws upon evolutionary biology, anthropology, animal behavior, physiology, neuroscience, linguistics, and other fields, many of which also overlap to varying degrees with comparative psychology while not being the same as comparative psychology. It probably is true that the typical undergraduate best equates the idea of comparative psychology with approaches in comparative cognition (or, perhaps, depending on the department, with Pavlovian and Skinnerian learning theories). A curriculum that emphasizes Learning and Behavior and Cognitive Psychology courses, along with an interdisciplinary approach with Biology and Neuroscience could work toward a resolution of this concern even if a course in Comparative Psychology did not exist.

We agree that three of the most critical issues facing undergraduates who might consider graduate training in comparative psychology are (1) access to animal research as undergraduates, (2) the sense that animal research is under increasing threat from animal rights advocates and (3) limited career opportunities, especially in academia, for those trained in comparative psychology. The first problem can be overcome by looking for opportunities outside university and college settings. Zoos, sanctuaries, and other such organizations that have animals can and will accept motivated interns. In fact, we encourage this approach, because one thing it offers that many comparative psychology students of last century largely did not get is the idea that animal behavior means more than the behavior of rats and pigeons! Comparative psychology needs to be *more comparative* (Beran et al., 2014), a problem that was a *greater* concern when comparative psychology was more prominent as an area of focused training (e.g., Beach, 1950). Going to where the animals are could help with this problem, and it already has in the sub-area of comparative cognition (e.g., Shettleworth, 2009). Of course, this also requires faculty that support such efforts by interested students. Concerns about animal rights activists can be addressed by teaching undergraduate students about the ways in which psychological scientists ethically and responsibly work with animals, with discussions about IACUC protocols, and with information about organizations such as APA's CARE committee. Finally, jobs in academia tend to go “where the money is,” and so this may require a bottom-up approach where re-establishing a stronger pipeline of students trained in the tradition of comparative psychology and successful in generating grant support then generates a larger workforce that can train future generations in the same tradition. Certainly, one can look at areas such as neuroimaging research to see how this trend emerges. At the same time, jobs for comparative psychologists may take other names, and even come from non-psychology departments (e.g., evolutionary biology), but they still exist and still require students trained in the methods of comparative psychology.

Comparative psychology remains a crucial and necessary aspect of a full psychological theory of behavior, including human behavior, and it needs to be presented to undergraduate students as such. More can be done, and it should, but in the end we think that there is less of a crisis than Abramson (2015) suggests. We do see a current lethargic promotion of the breadth of topics in a formally-defined comparative psychology (excluding the area of comparative cognition), but at the same time there is a strong and growing promotion of interdisciplinary approaches to animal behavior and cognition. Thus, “comparative psychology” as a distinct training tradition may fade, but if students still are exposed to rigorous methods for studying animal (and human) behavior in traditions such as comparative cognitive science, developmental science, biological science, or neuroscience, “comparative psychology” as a field is alive and well and still highly attractive to future students.

## Funding

Writing of this article supported by NICHD grant HD060563.

### Conflict of interest statement

The authors declare that the research was conducted in the absence of any commercial or financial relationships that could be construed as a potential conflict of interest.
